# What are the effects of pilates and dance on upper limb functionality in women post-breast cancer surgery? a randomized three-arm clinical trial

**DOI:** 10.1007/s00520-026-10362-1

**Published:** 2026-02-12

**Authors:** Leonessa Boing, Tatiana de Bem Fretta, Mirella Dias, Alcyane Marinho, Brigid Lynch, Anke Bergmann, Juliana da Silveira, Larissa Altenhofen Groth, Adriana Coutinho de Azevedo Guimarães

**Affiliations:** 1 Center of Health and Sport Sciences, State University of Santa Catarina, Florianópolis, Brazil; 2 Faculty of Medicine of Ribeirão Preto, University of São Paulo, Ribeirão Preto, SP Brazil; 3 Cancer Epidemiology Division, Cancer Council Victoria, Melbourne, Australia; 4 Clinical Epidemiology, National Institute of Cancer, Rio de Janeiro, Brazil; 5 Pascoal Simone, 358 – Coqueiros. 88080350, Florianopolis, SC Brazil

**Keywords:** Range of joint motion, Dance, Muscle strength, Breast neoplasm, Exercise and movement techniques

## Abstract

**Objective:**

To evaluate the short-term (16 weeks post-intervention) and long-term effects (six- and 12-month follow-up) of two physical exercise interventions—Mat Pilates or belly dance—compared with a control group on lymphedema, range of motion, isometric strength, proprioception, and upper-limb symmetry in women who have undergone unilateral breast cancer surgery and are receiving hormone therapy.

**Methods:**

Sixty-nine women who underwent unilateral breast cancer surgery were randomized into three groups: Mat Pilates, belly dance, and control. The Mat Pilates and belly dance groups participated in supervised exercise sessions three times per week, lasting 60 min each, for 16 weeks. The control group continued their usual daily activities and attended three educational lectures. The outcomes assessed included upper-limb functionality (DASH), lymphedema (arm volume), range of motion (digital goniometer), isometric strength (dynamometer), and limb symmetry (difference between the contralateral and homolateral limbs).

**Results:**

In the Mat Pilates group, post-intervention effects were observed in increased range of motion (p = 0.026), improved isometric strength (p = 0.001), and enhanced strength symmetry between limbs (p = 0.034). In the belly dance group, post-intervention improvements were identified in upper-limb functionality (p = 0.001), lymphedema (p = 0.017), isometric strength (p = 0.001), and symmetry of both range of motion (p = 0.041) and strength between limbs (p = 0.009). However, none of these gains were sustained at follow-up assessments. No significant intragroup changes were observed in the control group.

**Conclusion:**

Mat Pilates and belly dance were effective in improving upper-limb functionality, range of motion, lymphedema, isometric strength, and limb symmetry in breast cancer women undergoing hormone therapy. Both forms of physical exercise may be recommended by professionals involved in oncological rehabilitation.

**Registry:**

ClinicalTrials.gov (NCT03194997) Registration June 20, 2017.

**Supplementary Information:**

The online version contains supplementary material available at 10.1007/s00520-026-10362-1.

## Introduction

Despite advances in surgical techniques for the treatment of breast cancer, a series of side effects on the functionality of the upper limb homolateral to surgery are still expected [1, 2], the main ones being: decreased range of motion, decreased strength, change in proprioception, and asymmetry of strengths and range of motion between upper limbs [3–5]. In addition, one of the most feared complications after breast cancer surgeries is lymphedema [6], which can alter mobility and hinder the activities of daily life [3]. In this sense, there is an important need for therapeutic approaches that can mitigate these side effects and assist in the recovery of these women.

Systematic reviews [4, 7, 8] have provided consistent and robust evidence regarding the benefits of physical exercise, highlighting significant improvements in the function, mobility, and range of motion of the upper limbs. Furthermore, exercise has demonstrated a crucial role in the prevention of breast cancer-related lymphedema, further reinforcing its importance in recovery and post-surgical management of the disease. Another notorious aspect is that, despite the benefits added to physical exercise, most women with breast cancer do not reach the recommendations for physical activity, and in turn, for physical exercise defined by the World Health Organization (WHO) [9].

In this context, exercise modalities that integrate body and mind, such as the Mat Pilates and belly dance, have emerged as promising strategies in oncological rehabilitation. The Pilates method combines resistance and stretching exercises synchronized with breathing and guided by the principles of control, precision, concentration, fluidity, and core stabilization [10]. Evidence indicates that Pilates can improve range of motion, strength, proprioception, and postural control, contributing to the recovery of neuromuscular coordination and functional symmetry in women after breast cancer treatment [11, 12].

Similarly, dance movement interventions, including belly dance and Dance Movement Therapy (DMT), promote rhythmic and coordinated movements involving the upper limbs, trunk, and pelvis, which may enhance proprioceptive feedback, body awareness, and motor control [13]. Although studies specifically investigating belly dance in women with breast cancer are scarce, findings from broader dance interventions indicate potential benefits for mobility, balance, proprioception, and functional recovery [14, 15]. These characteristics suggest that both modalities may influence proprioception and other parameters commonly impaired after breast cancer surgery; however, the evidence remains limited and inconsistent, especially regarding long-term effects and direct comparisons between modalities.

Thus, considering the high incidence of breast cancer and the common post-treatment impairments in upper limb functionality [4, 16, 17], alternative physical exercise modalities such as the Mat Pilates method and belly dance may represent interesting and feasible strategies to enhance adherence and engagement in physical activity among these women. However, although preliminary findings suggest that both Pilates [12, 13] and dance-based therapies [14, 15] may influence upper limb function, the evidence remains limited and inconsistent, particularly regarding long-term outcomes and comparisons between these two modalities in breast cancer survivors. This gap highlights the need for studies assessing their specific effects on proprioception, strength, arms symmetry, and lymphedema.

The objective of this study was to analyze the short-term (post-intervention) and long-term (6- and 12-month follow-up) effects of a 16-week intervention with physical exercise (Mat Pilates or belly dance) on lymphedema, range of motion, isometric strength, proprioception, and upper-limb symmetry in women undergoing hormone therapy treatment after unilateral breast cancer surgery.

## Materials and methods

### Design of the study

This study is part of the MoveMama project, which is a prospective, single-center, three-arm randomized clinical trial developed according to the CONSORT 2010 (Consolidated Standards of Reporting Trials) standards at the Center for Oncological Research (CEPON). The details of the research protocol were previously published [18]. The study was approved by the Research Ethics Committee (CEPSH) of UDESC, protocol number 2252288 and by the IRB of CEPON (CEP), protocol number 2.319.138. The study was registered on the ClinicalTrials.Gov platform (NCT03194997) on June 20, 2017, and was carried out in accordance with the Helsinki Declaration [19].

### Participants

Women were recruited at CEPON, from a list of hormone therapy users between 2015 and 2017. Inclusion criteria were: (1) women aged 18 years or older; (2) diagnosed with breast cancer in clinical stages I to III; (3) currently undergoing hormone therapy; (4) had completed surgical treatment (mastectomy or unilateral conservative surgery); (5) had medical clearance to participate in physical activity; and (6) resided in the metropolitan area of Florianópolis or São José (Southern Brazil). Exclusion criteria were: (1) physical or neurological limitations that could prevent participation in the exercise sessions (e.g., Parkinson’s disease, Alzheimer’s disease, or use of a wheelchair); (2) presence of metastatic disease; (3) ongoing radiotherapy or chemotherapy treatment; and (4) bilateral surgery.

Five women were excluded from the analyses because they had undergone bilateral surgery. This decision was made because the analysis aimed to evaluate the effects of the intervention on the affected limb versus the contralateral limb. To ensure sample homogeneity and reduce potential bias, only patients who had undergone unilateral surgery were included.

Randomization was stratified by age and performed through a site (http://www.randomization.com) by two researchers (LB and TBF), in order to allocate women in three groups: intervention with Mat Pilates method; intervention with belly dance; and control group. Due to the nature of the interventions and the limited size of the research team, blinding of participants, intervention providers, outcome assessors, and data analysts was not feasible. We acknowledge this as an inherent limitation of behavioral and exercise-based trials. To minimize the potential influence of this non-blinding on the study results, all assessments followed strict standardized protocols, conducted using objective measurement instruments with predefined procedures to reduce subjective interpretation. Additionally, data entry and statistical analyses were performed following a prespecified analysis plan, minimizing analytic bias. Although assessors and analysts were aware of group allocation, the use of objective outcomes and standardized procedures helps reduce the risk of systematic measurement bias.

### Interventions

All sessions took place at CEPON during the 16 weeks of intervention, at a frequency of three times a week, with classes of 60 min in the morning. Each session was divided into (i) warm-up and stretching, (ii) main part of the class and (iii) relaxation. The exercises were performed according to the capacity of each participant, respecting the principle of biological individuality [20] with the intention of causing positive adaptations to exercise, avoiding psychological embarrassment and emotional difficulties.

The 48 sessions of Mat Pilates method and belly dance are detailed in the protocol of the previously published study [18]. Briefly, for the Mat Pilates method, conducted by a physiotherapist with over ten years of experience, the sessions were performed in groups, but each exercise was executed individually and focused on upper and lower limb flexibility, joint mobility, core strengthening, and breathing control. Most exercises were performed in the supine position to avoid joint overload, and the intensity was progressively increased using resistance bands, magic circles, and toning balls. Examples include trunk rotation with upper limb abduction, leg stretches with elastic resistance, and abdominal stabilization exercises. After the 10th session, a tonic ball of 500 g was added to trunk rotation exercises associated with upper limb abduction; in the 20th session, an elastic band was incorporated into upper limb circle exercises; and after the 24th session, the same trunk rotation exercise was performed with a 1 kg load.

For the belly dance intervention, instructed by a Physical Education professional with more than ten years of dance experience, sessions emphasized motor coordination, rhythm, body awareness, flexibility, and range of motion of the upper limbs, incorporated into choreographed dance routines. The movements involved ondulations, hip circles, shoulder shimmies, and veil work, and were performed individually, but the classes included group dynamics in circles and pairs to stimulate interaction and engagement. The intensity of the dance classes was progressively adjusted according to the rhythm of the music, controlled through beats per minute (bpm): warm-up (80–120 bpm), main session (120–150 bpm), and cool-down (≤ 80 bpm) [21]. In the warm-up session were used songs from 80 to 120 bpm, in the main part songs from 120 to 150 bpm, and in the back session the calm/relaxation was used songs of up to 80 bpm. These bpm values correspond exclusively to the tempo of the songs used to guide the progression and intensity of the dance sequences. Participants’ heart rates were not measured, as bpm refers only to musical rhythm and not to physiological monitoring.

Detailed tables describing all exercises, progressions, and dance movements performed during the 48 sessions are available in the Supplementary Material.

Three educational lectures were offered to the control group in the first week, in the 8th week and in the 16th week in the hospital auditorium. Each educational session addressed a different theme: (i) the first was taught by a physiotherapist and a physical education professional, and focused on the description of stretching exercises to be performed at home; (ii) the second session, given by a breast cancer patient, addressed advice on how to deal with self-esteem and body image after breast cancer, and (iii) the last meeting was given by a physiotherapist and focused on changes in behavior in relation to the prevention of lymphedema.

At the end of the 16 weeks of intervention, all women received an educational booklet containing recommendations for physical activity and information about breast cancer and a t-shirt from the MoveMama study. They were also invited to be part of a free extension program involving physical activity for breast cancer survivors at the University of the State of Santa Catarina (UDESC).

### Data collection

Data collection occurred in the baseline period of the study, in the post-intervention, and in the follow-up of six months and 12 months. Data collection took place from March 2018 to December 2019, and each evaluation lasted an average of 60 min.

### Personal and clinical information

Personal information included age, nutritional status and physical therapy in the baseline period. In turn, the clinical information consisted of modality of hormoniotherapy, previous clinical treatment, side of the surgery, characteristics of the surgery, time after surgery, breast reconstruction, axillary emptying. This information was evaluated in the baseline period of the study through a questionnaire applied by two researchers (LB and TBF) during an interview with each participant. Height (Sanny wall stadiometer, American Medical Ltda, Brazil) and body mass (Toledo 2096 PP digital scale, Toledo Industry Ltda, Brazil) were evaluated to identify body mass index (BMI), which was calculated (kg/m2) and classified according to WHO in normal weight (18.5—24.9 kg/m2) and overweight (25.0 kg/m2).

### Outcome variables

#### Functionality of the upper limb

The functionality of the upper limb in this study is represented by the variables of functionality of the arms, shoulders and hands, range of motion, isometric strength, proprioception and lymphedema. All these variables were evaluated in the four study periods: baseline, post-intervention, follow-up of six and 12 months.

The functionality of the arms, shoulders and hands was evaluated using the instrument Disabilities of the Arm, Shoulder and Hand (DASH) [22], validated in Brazil [23]. This is a questionnaire of 30 items focusing on the functionalities and symptoms of the upper limb. DASH scores range from zero to 100, and a higher score indicates worse upper limb functionality.

The active range of motion of the shoulder was evaluated by means of a digital goniometer Absolute Axis 360º (Baseline, United States), in the positions of flexion, abduction and external rotation of the shoulder of the affected and unaffected arm. For the position of shoulder abduction the women were seated in a chair, the feet supported on the floor, and for the position of flexion and external rotation of the shoulder the participant was lying on the stretcher [24].

The maximum isometric strength of the homolateral and contralateral arms was evaluated by means of a portable dynamometer Chatillônₒ (Ametek, United States). The test was performed in five positions: shoulder flexion, shoulder extension, shoulder abduction, external rotation of the shoulder and internal rotation of the shoulder. For each test position, the participant was lying on the stretcher and was instructed to make maximum strength against the dynamometer for seven seconds. Three repetitions were performed for each position with an interval of 30 s between each attempt. The average of the three repetitions was used for the final analysis of the data and presented in Newtons.

Proprioception was evaluated by means of a repetition of the articular position in a kinesiometer, validated in Brazil [25]. The kinesiometer ranges from 0 to 180 degrees and the test consists of moving a wooden movable arm in three positions (90 degrees, 45 degrees and 105 degrees). The participant performed the test sitting in a chair, blindfolded, feet supported on the floor, with a table in front of her containing the kinesiometer and the arm in dorsal decubitus under the mobile arm of the kinesiometer. The researcher first moves the participant’s arm in the three positions, holding for 10 s in each position. Then, the participant is asked to perform the movement of the movable wooden arm in the three positions without the help of the researcher. The degree of each joint position is recorded and then decreased by the original degree, demonstrating how many degrees were missing or exceeded the exact point. The degrees of error of the three positions are summed and divided by three to provide an absolute error.

The calculation of lymphedema was performed by calculating the volume of the arm, performed by measuring the circumferences of both upper limbs, in five points distributed along the arm and forearm: 21 cm and 11.5 cm above the olecranon; at 7.5 cm, 14 cm and 24 cm below the olecranum. The circumference was obtained, with the patient seated, keeping the arm in abduction, flexed forearm and the hand resting on the chest. These measurements were used to calculate the approximate volume of the five truncated cones, formed at the points of measurements of the circumferences. The sum of these five parts gives the total volume of the limb and the difference between the limbs represents the presence of lymphedema, if it is greater than 200 ml [26].

A symmetry analysis of range of motion and isometric strength was conducted to determine whether the intervention altered the symmetry between the ipsilateral and contralateral limbs to the surgery in the post-intervention period. In the range of motion, the values of the contralateral limb were subtracted from the values of the affected limb [27]. The formula (2*(Strength contralateral side—Strength homolateral side)/(Strength contralateral side + Strength homolateral side)) *100% [28] was used to calculate the symmetry for strength.

### Sample size and statistical methods

For this study, the calculation was performed in the software G.Power 3.1.9.7 [29], considering the alpha (α) of 0.05, power (1-beta) of 0.80, and the effect size (F2) of 0.22, a median effect [30]. Based on the three study groups and the four data collection moments (baseline, post-intervention, six months and 12 months), 39 participants would be needed. Including a sample loss of 30%, 51 participants would be needed, with 17 in each group.

Data were expressed in frequency, mean, standard deviation and/or standard error. Comparison of categorical results between groups (Mat Pilates method, belly dance and control) at baseline was performed by Chi-square test or Fisher’s exact test (n < 5). After the normality test with the Kolmogorov Smirnov test, one-way ANOVA was used to compare age between the three groups and the Kruskal–Wallis test for comparison of months after surgery.

According to the protocol study Boing et al. [18], comparisons between baseline and post-intervention between the groups method Mat Pilates, belly dance and control the two-way ANOVA test with repeated measures and LSD comparison test was used. An intention-to-treat analysis was used and included all participants who completed the baseline period and data collection in the post-intervention, regardless of adherence to the intervention. Additionally, protocol analysis was included with women who completed 50% of the sessions, and this table is presented as supplementary material, since the effects were similar.

For long-term effect analysis, when considering the loss of follow-up, we chose to use the Generalized Estimating Equation (GEE) for follow-up analysis between the groups that received intervention with physical exercise (Mat Pilates and belly dance method). This analysis was performed by intention to treat, including all women who participated in the study in the baseline period, and completed follow-up collections. It is noted that the control group was not included in this analysis due to high sample loss at follow-up, and therefore, small sample in the six (n = 7) and 12 months (n = 5) in these variables. To verify the effects of change between symmetries, the two-way ANOVA test with repeated measures and LSD comparison test was used and the results were presented in a graphic format, built by GraphPad Prism 6. All analyses of this study were performed on IBM SPSS Statistics software version 20.0 (IBM, Armonk, NY, USA). A significance level of p < 0.05 was considered.

## Results

### Recruitment and flow chart of participation

**Fig. 1:**
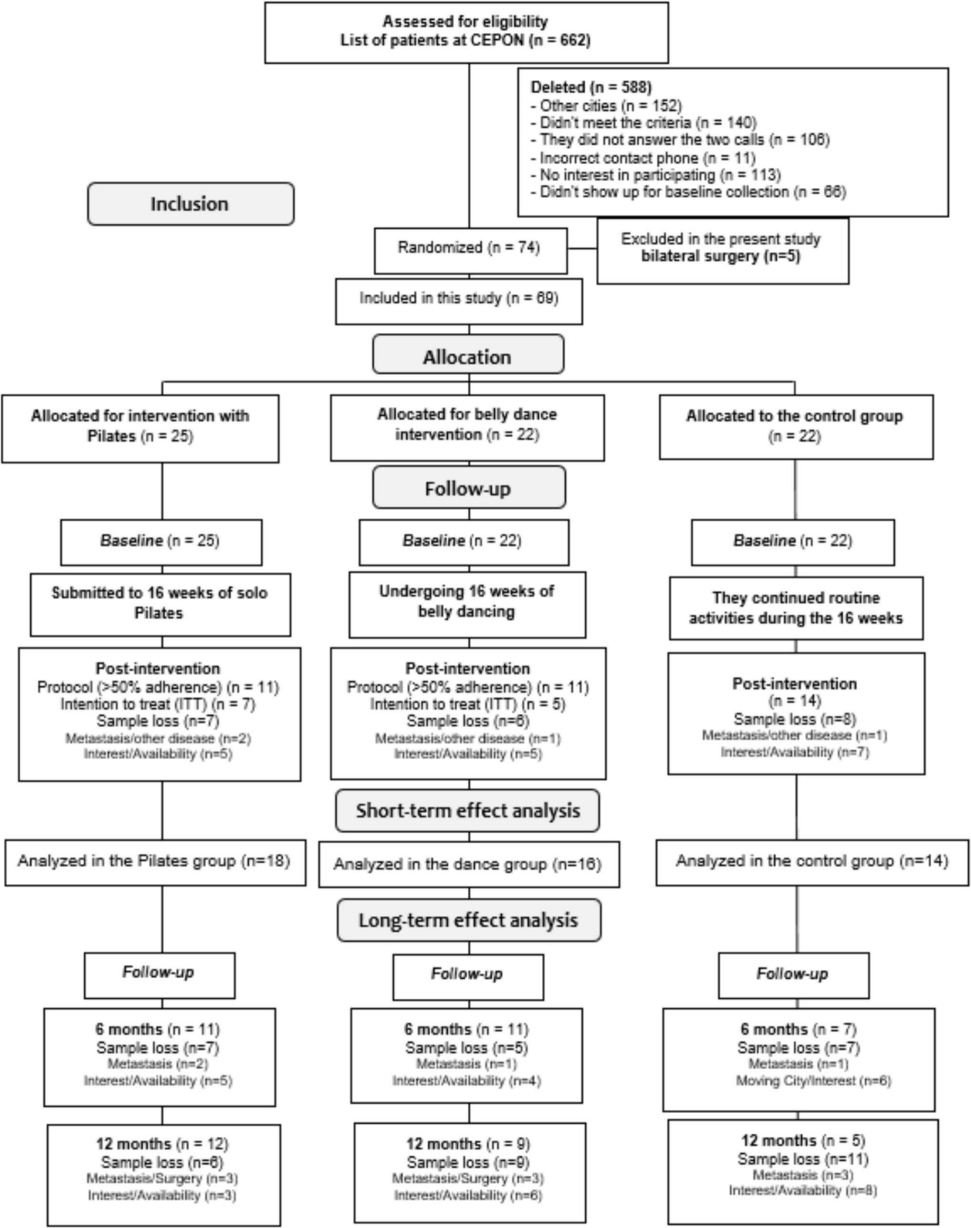
Flowchart for selecting study participants. Source: Prepared by the author (2021)

We included 74 women undergoing hormone therapy in CEPON, randomized in the three study groups. Of these, five were excluded because they had undergone bilateral surgery. During the 16 weeks of intervention, 22 patients (30%) did not complete the follow-up, being included in the short-term analysis, in the six-month follow-up, 29 women were analyzed and, in 12 months, 26 women (Fig. 1).

### Characteristics in the baseline period

No differences were observed between personal and clinical characteristics in the baseline period between the study groups, ensuring randomization and homogeneity of the sample. Overall, the 69 women included (55 ± 10 years of age) were 29 ± 18 months after unilateral breast cancer surgery, and most of them underwent conservative surgery (65%), on the right side (54%), with lymph node dectomy approach (57%); and among those submitted to mastectomy, 52% of women underwent breast reconstruction (52%). Still, most of the women in the study were overweight (67%) according to body mass index, were using aromatase inhibitors as a hormone therapy (59%) and was not receiving physical therapy at the time of the study (94%) (Table as supplementary material).

### Intervention effects

Table 1 shows the main short-term effects of the intervention groups compared to the control group on shoulder functionality, lymphedema, range of motion, isometric strength and proprioception, by intention to treat between baseline and post-intervention periods. When observed the functionality of the upper limb, through the DASH questionnaire, a significant intra-group improvement was observed for the belly dance group (Δ = −21; p = 0.001), as well as for lymphedema, with intragroup effect for belly dance (Δ = −28 ml; p = 0.017) representing a smaller difference between the two extremities in the post-intervention period.

**Table 1:** Short-term effect (baseline versus post-intervention) of interventions with Pilates method, belly dance and control group in the variables of upper limb functionality by analysis of intention to treat

	Mat Pilates Group (n = 18)			Belly Dance Group (n = 16)			Control group (n = 14)			
	*Baseline*	Post intervention	Intragroup effect p value*	*Baseline*	Post intervention	Intragroup effect p value*	*Baseline*	Post intervention	Intragroup effect p value*	Intragroup effect p value#
Upper limb functionality (DASH)	41(4)	37(4)	0,426	58(4)	37(4)	**0,001**	43(5)	33(5)	0,096	0,205
Difference in limb volume (ml)	−21(52)	−7(44)	0,843	103(52)	−75(44)	**0,017**	35(55)	−58(47)	0,233	0,788
*Homolateral side to the surgery*										
ADM (^o^)										
Abduction	145(8)	155(6)	0,360	140(8)	157(6)	0,105	147(9)	152(7)	0,673	0,966
Flexion	148(7)	170(6)	**0,026**	144(7)	156(6)	0,192	147(8)	157(6)	0,346	0,425
Rotation	76(5)	86(4)	0,179	75(5)	86(4)	0,127	84(5)	83(4)	0,830	0,136
Strength (N)										
Flexion	80(9)	109(11)	**0,023**	70(10)	106(12)	**0,006**	80(11)	87(12)	0,574	0,689
Extension	72(8)	108(9)	**0,001**	63(9)	101(10)	**0,001**	74(9)	82(10)	0,454	0,098
Abduction	78(9)	101(10)	0,052	62(10)	103(11)	**0,002**	71(10)	74(11)	0,786	0,125
Internal Rotation External Rotation	58(6)	72(6)	0,060	45(7)	72(7)	**0,002**	46(7)	56(7)	0,201	0,180
	53(6)	70(6)	**0,036**	45(7)	59(6)	0,120	52(7)	54(6)	0,819	0,342
Proprioception (^o^)	27(3)	19(3)	0,097	20(3)	21(3)	0,816	27(4)	21(3)	0,263	0,569

Legend: Anova two-way with repeated measures and comparison of minimal significant difference (LSD). * p value for intragroup comparison between baseline and post-intervention (Effect of interaction between group versus time). # the value for intergroup comparison between baseline and post-intervention (Isolated group effect). ADM, Range of motion. ml, mm. o, degrees. N, Newtons. Values expressed by means of the mean (standard error)

Regarding the changes in the upper limb of the homolateral side to surgery, there is an effect in the post-intervention on improving the range of motion in intragroup flexion for the Mat Pilates method (Δ = + 22o; p = 0.026). With regard to the isometric strength of the homolateral side to surgery, the group submitted to the intervention with the Mat Pilates method showed significant intragroup effect with improvement in flexion (Δ = + 29 N; p = 0.023), extension (Δ = + 36 N; p = 0.001) and external rotation (Δ = + 17 N; p = 0.036). Likewise, the belly dance group showed significant intra-group effect with improvement for flexion (Δ = + 36 N; p = 0.006), extension (Δ = + 38 N; p = 0.001), abduction (Δ = + 41 N; p = 0.002) and internal rotation (Δ + 27 = p.002). The control group did not show any significant intragroup change. No significant change was observed for proprioception in the upper limb of the homolateral side to surgery.

**Fig. 2:**
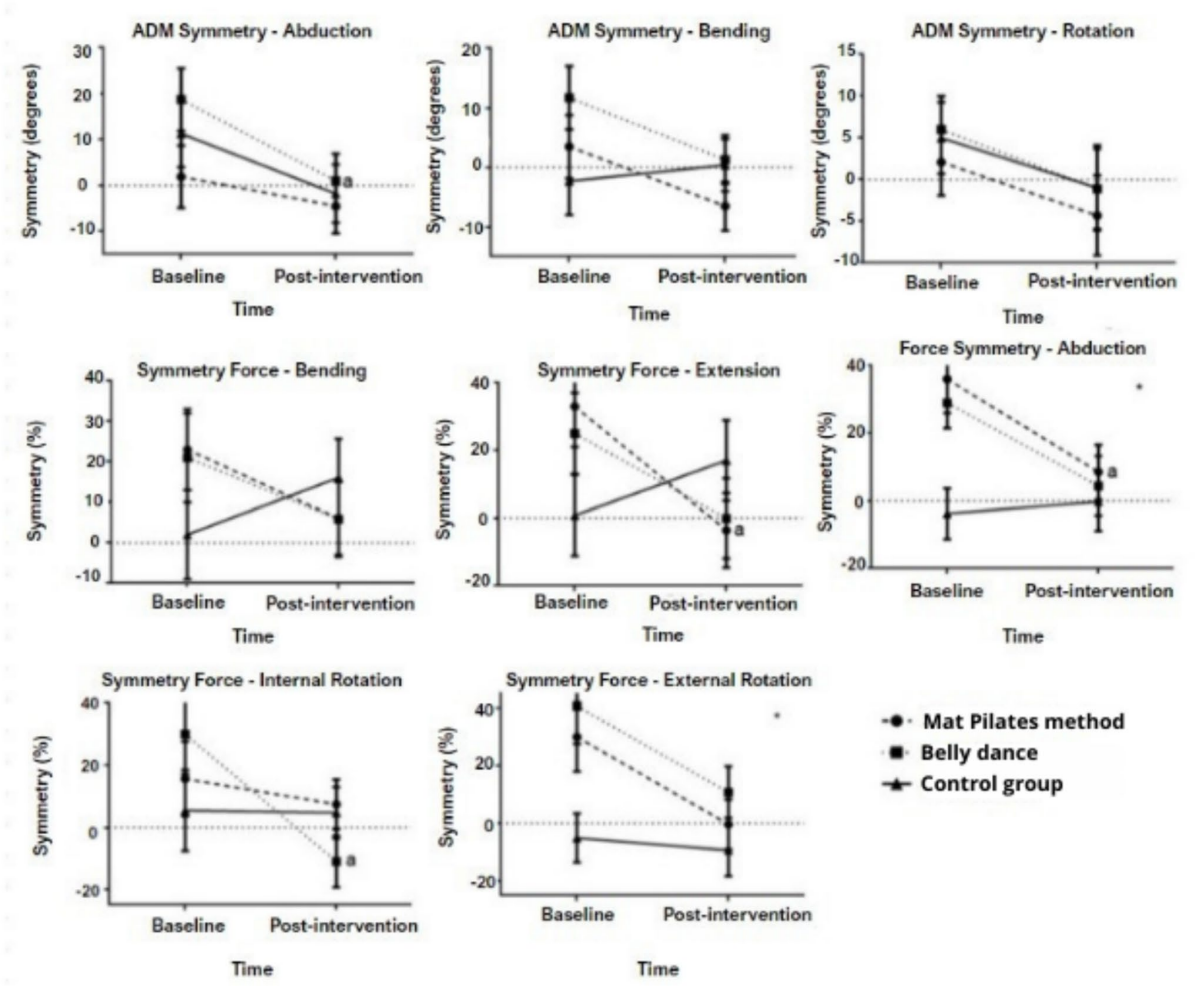
Short-term effect of physical exercise intervention (Mat Pilates method or belly dance) between baseline and post-timeintervention for the symmetry of the range of motion and isometric strength between the homolateral and contralateral limbs to the surgery of the study participants.. Legend: ROM, range of motion, (*) represents significant intergroup effect (p < 0.05), (**a**) represents significant intragroup effect (p < 0.05). Analysis performed by two-way Anova with repeated measures and LSD comparison test

Figure 2 shows the effects of the intervention on the symmetry of the range of motion and isometric strength between the homolateral and contralateral limbs at surgery for the three study groups (Mat Pilates method, belly dance and control). One can observe an intragroup effect for the belly dance group in the symmetry of the range of motion of the abduction, with improvement after the intervention (18th to 1º; p = 0.041). In isometric strength, an intragroup effect was found for the Pilates soil group, with improved symmetry between the limbs for extension (33% for −4%; p = 0.034), and improved symmetry between the limbs in abduction (36% for 8%; p = 0.044). Also, an intergroup effect was found for abduction (p = 0.031), with difference between the Mat Pilates versus control group (p = 0.011). In the isometric strength of internal rotation, an intragroup effect was found for the belly dance group (30% for −11%; p = 0.009). In the external rotation, an intergroup effect was identified (p = 0.010), with difference in symmetry between the Mat Pilates group versus control group (p = 0.030) and belly dance group versus control group (p = 0.003).

**Fig. 3:**
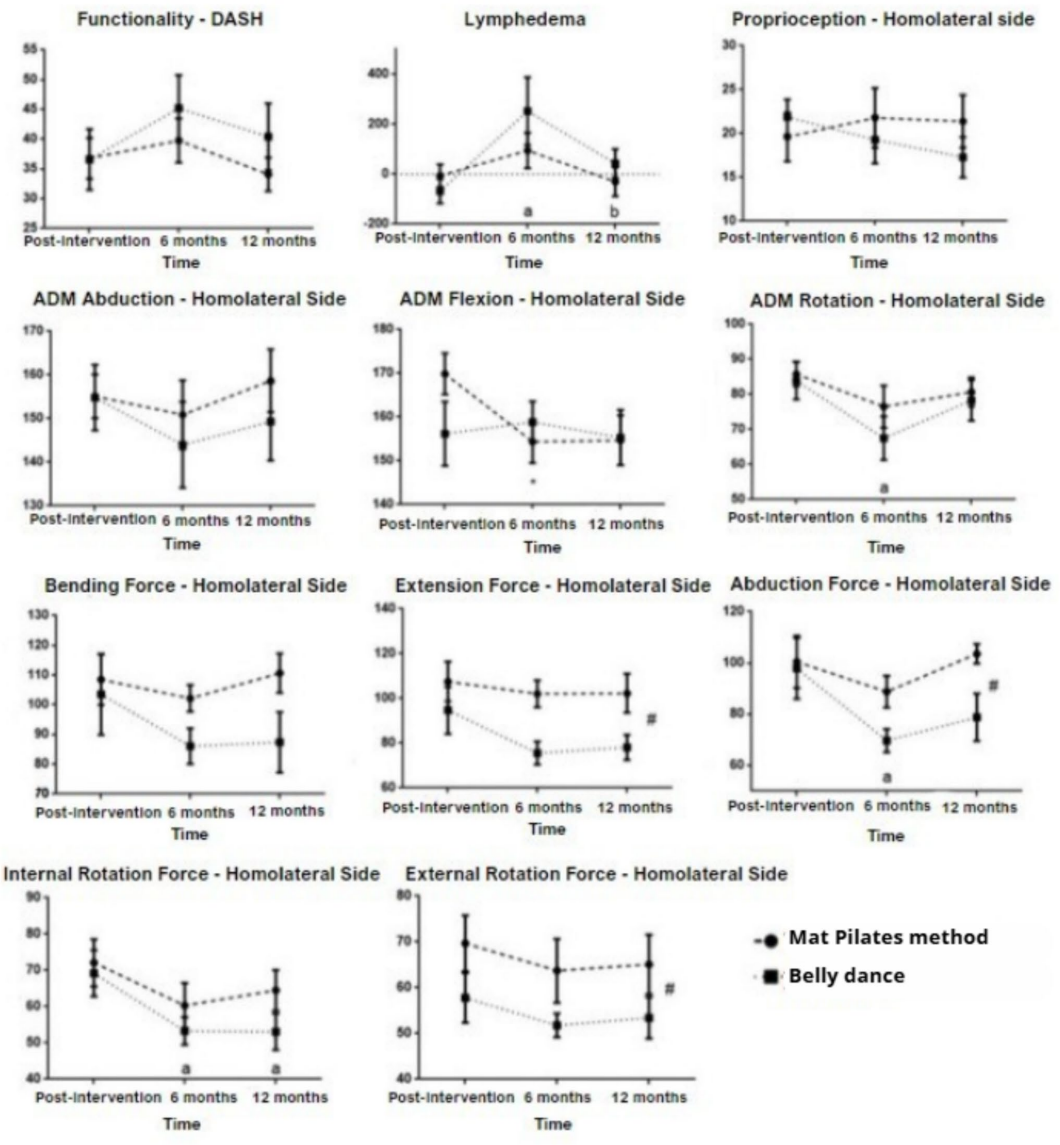
Effects of physical exercise intervention (Mat Pilates method or belly dancing) btween post-intervention and six- and 12-month follow-up times for upper limb functionality (DASH), lymphedema (difference in arm volume), proprioception, range of motion (ROM), and isometric strength of the upper limb on the ipsilateral side of the surgery

Figure 3 shows the effects on upper limb functionality (DASH), lymphedema, proprioception, range of motion and isometric strength of the upper limb of the homolateral side to surgery, in relation to changes between post-intervention and follow-upup of six and 12 months, comparing only the groups that received intervention with Mat Pilates method and belly dance. There were no significant changes in DASH and proprioception. In lymphedema was perceived a significant effect of time (p = 0.032), between the post-intervention and follow-up of six months with increased difference between limb volumes for both groups (Pilates Δ = −101 ml; dance Δ = −319 ml; p = 0.010) and again decreasing the difference between the volumes between the follow-up of six and 12 months (Pilates Δ = + 125 ml; dance Δ = + 209 ml; p = 0.018). In the range of motion, a decrease in flexion movement was observed between the post-intervention and the six months of follow-up only for the Pilates group (Δ = −15o; p = 0.044), and for the rotation movement in both groups in this same period (Pilates Δ = −9o; dance Δ = −16o; p = 0.025).

In the isometric strength, a decrease was observed during the follow-up, in which in the abduction movement, both groups presented a decrease between the post-intervention and the follow-up of six months (Pilates Δ = −12N; dance Δ =—28N; p = 0.012); and in the internal rotation movement a decrease between the post-intervention and the follow-up of six months (Pilates Δ = −12N; dance Δ = −16N; p = 0.004), and between the post-intervention and the follow-up of 12 months (Mat Pilates Δ = −7N; dance Δ = -N = -N = -N; N = -N = -N). One can also perceive in the isometric strength a significant difference between groups, with higher values for the Mat Pilates method in the extension movement (p = 0.003), abduction (p = 0.026) and external rotation (p = 0.035) in the follow-up period.

Legend: (a) represents significant effect of time in relation to the post-intervention for both groups, (*) represents significant intra-group effect, (#) represents intergroup difference. Analysis performed using the Generalized Estimating Equation, controlled by the modality of surgery (mastectomy or conservative surgery), values expressed in average and standard error.

## Discussion

It was observed that the Mat Pilates group had short-term positive effects on the improvement of the range of motion of flexion, increase of isometric strength in flexion, extension and external rotation, and symmetry of isometric strength for extension and abduction; In turn, the belly dance group demonstrated short-term effects on improved functionality of the upper limb, decreased lymphedema, increased isometric strength in flexion, extension, abduction and internal rotation, as well as, an improvement in the symmetry of the limbs in the range of motion of the abduction and in the symmetry of the isometric strength of internal rotation. Both groups were effective in the short term to improve aspects of the functionality of the upper limb of women using hormone therapy. It is observed that in the short term the control group did not present significant effects.

The short-term improvements observed in the Pilates group may be explained by the method’s emphasis on controlled multi-planar movements, progressive overload using elastic bands and light weights, and activation of stabilizing musculature. These elements are known to induce neuromuscular adaptations that increase strength, improve scapulohumeral rhythm, and facilitate greater shoulder flexion [31, 32]. Similar mechanisms have been reported in previous trials examining Pilates in breast cancer survivors [33–35].

Improvements in symmetry and upper limb functionality in the belly dance group may be explained by the fact that this modality involves repetitive arm movements, performed in multiple directions and often alternating between sides. These movements help improve coordination, body awareness, and control of shoulder motion, which can contribute to greater symmetry and functional gains [36, 37]. Similar benefits have been reported in studies using dance-based or rhythmic movement interventions, which show positive effects on motor coordination, mobility, and upper limb function in women after breast cancer treatment [34].

When observed the maintenance of the positive effects obtained by the intervention in the follow-up period of six and 12 months, one can perceive a behavior of increased volume of the arms for both groups method Mat Pilates and belly dance, in the follow-up of six months and decrease in the 12 months, a decrease in the amplitude of movement in the flexion and rotation movement in the six months, and a decline in the isometric strength in the abduction movement in the six months and in the internal rotation in the six and 12 months. This worsening in these aspects in the follow-up period, specifically, increased lymphedema, decline in range of motion and isometric strength, demonstrate the importance of the practice of supervised physical exercise during the 16 weeks of intervention, to maintain healthy habits and return to activities of daily life. Also, this pattern is consistent with exercise detraining responses, reported in a systematic review involving healthy adults performing concurrent training, in which gains obtained through exercise tend to diminish within 2 to 4 weeks after cessation, depending also on the intensity of the interventions [38].

The findings regarding the benefits of the Mat Pilates group in improving the range of motion after 16 weeks of intervention are corroborated by a systematic review of the Pilates method in breast cancer [11]. Similarly, a umbrella review, pointed out a strong evidence that Pilates can be beneficial for increased flexibility, since this practice works stretching the upper limb dynamically [39]. Considering that the present study is the Mat Pilates method, easy to perform on the floor, these findings can help in oncological rehabilitation, enabling the exercise in small spaces, with the use of elastic band, magic circle, weights of 1 kg and Pilates ball taught by a professional in the area.

Regarding isometric strength, both groups, Mat Pilates and belly dance, demonstrated effects in the post-intervention when compared to the control group. Two other randomized controlled trials demonstrated similar effects on strength increase after twenty four weeks of Mat Pilates [33, 40] and a randomized clinical trial demonstrated improvement in strength after 16 weeks of Latin American dance [41]. A systematic review provides favourable evidence that aerobic exercise, including yoga and Pilates, can be an exercise option to help improve muscle strength after breast cancer surgery, even though the resistance exercise is normally recommended [42]. In the present study, the classes of the Mat Pilates method, were increased by the use of weight of 1 kg and elastic band, and the belly dance classes, had their choreographic sequences developed with use of handkerchiefs and repetitions of movements of the arms. These factors may have helped in the findings of improvement of isometric strength by the way movements are explored in both groups.

An interesting finding of the present study was in relation to the symmetry of range of motion and isometric strength. For the Mat Pilates group significant effects were found in the symmetry of the isometric strength for extension and abduction, in turn, the belly dance group improved the symmetry of the limbs in the range of motion of abduction and the symmetry of the isometric strength of internal rotation. After breast cancer surgery, an asymmetry between the homolateral and contralateral upper limbs is natural, and this can affect the activities of daily living, as well as the functionality of the upper limb [1]. During belly dance classes, arm movements were dynamic and performed independently between both limbs, allowing a better stimulus of freedom to improve symmetry and range of motion between the two limbs. For Mat Pilates, the exercises were always performed by both arms, with sequential repetitions, and the two limbs used the weight of 1 kg, magic circle, or elastic band, similarly, providing an improvement of symmetry for isometric strength. Thus, the physical exercise interventions of this clinical trial may be recommended, suggesting that when there is asymmetry for range of motion, belly dance can be an effective alternative, and when in the presence of asymmetry in relation to isometric strength Mat Pilates classes may be an option. A suggestion for further studies would be to complement the activities by the two modalities.

Although significant short-term effects were perceived in the belly dance group, in relation to long-term lymphedema, it was not observed the maintenance of this improvement, with a behavior of increase in six months for both groups. Similarly, a study of yoga [43] found no significant differences in upper extremity edema volume between the intervention and control groups, either at baseline or after 4 and 8 weeks of intervention. However, the study indicated significant improvements in quality of life and physical and emotional functioning after 8 weeks. These findings suggest that, despite some improvements observed in the short term, the effects of treatment on edema volume may not be sustainable in the long term. Similarly, an umbrella review [44] evaluated the evidence on therapies for lymphedema in breast cancer and found a prevalence of 42% 18 months after surgery. Furthermore, some of the risk factors for incidence are total axillary dissection, obesity or overweight, and physical inactivity. It was demonstrated, along with the findings of the present study, the need to maintain continuous physical activity to preserve these benefits.

One of the outcomes that did not show changes in the post-intervention was proprioception. Proprioception, defined as the perception of limb position in space, remains a variable that has not been extensively investigated in the breast cancer literature. It allows individuals to recognize the position of a limb even without visual input, due to mechanoreceptors located in the skin, muscles, tendons, and ligaments [45]. Working proprioception is extremely important, since it can act assisting in the quality of movement and functionality of the shoulder, being that a worse proprioception can be found among women with breast cancer when compared to a group of healthy women of the same age, and changes in proprioception of women with lymphedema [46]. Perhaps the interventions were not effective for this variable due to the nature of proprioceptive feedback, which relies on both joint and cutaneous mechanoreceptors and therefore may require more specific activities to be properly stimulated [47]. In addition, further research is needed to clarify the extent to which proprioception is affected after breast cancer surgery and to determine the most effective strategies to improve it.

When considering the effects of this randomized clinical trial, it is important to highlight some limitations, such as the use of arm volume to identify lymphedema, which is suggested to be avoided in studies with exercise [48], because the increase in strength for example, can influence arm volume and confuse findings. Also, the inclusion of women submitted to conservative surgery as much as mastectomy, without differentiating the axillary approach may be limiting. However, there was no difference between these characteristics in the baseline between the randomized groups. Finally, one of the main limitations of the study was the sample loss during follow-up, which made it difficult to interpret the results, and led to the non-inclusion of the control group in the follow-up, also making it impossible to analyze the intention of treatment[49].

It is also important to list the strengths of the study, as the methodological design, being a randomized clinical trial with follow-up of up to 12 months, all instruments used are validated and addressed in the literature on breast cancer women, and the originality of the study, since it is an innovative comparison between the effects of Mat Pilates and belly dance in aspects of upper limb functionality in breast cancer women.

Still, the findings of this study may have impacts on the incorporation of new practices in hospital units, or even on the initiative of public policies adjusted to a new model of care that encompasses these practices. It is clear that the two interventions with physical exercise for 16 weeks performed in this randomized clinical trial, Mat Pilates method and belly dance, were effective in aspects of the functionality of the upper limb, and its application in oncological rehabilitation for women undergoing unilateral breast cancer surgery may be recommended.

### Limitations

This study has some limitations. A major limitation is the loss of participants in long-term follow-up, which limited the ability to analyze long-term effects in the control group. Additionally, the research team was small, and the same researcher delivered the interventions and conducted the assessments, which may increase risk of measurement bias even when standardized protocols were followed.

### Clinical Implications

Despite these limitations, the study offers relevant clinical implications. Both Mat Pilates and belly dance are low-cost, accessible, and adaptable modalities that can be incorporated into oncological rehabilitation programs. Since individual classes are not feasible in all clinical settings, group-based structured sessions with standardized protocols—similar to those applied in this trial—may serve as a practical alternative. Clinicians may also choose the modality based on the primary deficit: Pilates when strength and flexion deficits predominate, and belly dance when asymmetry and functional mobility require greater emphasis.

## Conclusion

In women with breast cancer undergoing adjuvant hormone therapy, short-term benefits were observed for both exercise interventions. The Mat Pilates group demonstrated improvements in range of motion, isometric strength, and upper-limb symmetry, while the belly dance group showed gains in upper-limb functionality, reduced lymphedema, increased isometric strength, and improved symmetry. However, these effects were not maintained at six and twelve months in either group, indicating that continuous or periodic exercise may be necessary to sustain the improvements. No short-term effects were found in the control group. Although the intervention was feasible as a group-based program, its implementation may vary across clinical settings, highlighting the need for standardized, accessible protocols to guide clinical practice.

## Supplementary Information

Below is the link to the electronic supplementary material.

Supplementary file1 (DOCX 15 KB)

Supplementary file2 (DOCX 571 KB)

## Data Availability

No datasets were generated or analysed during the current study.
