# Supplementary material for: What are the effects of pilates and dance on upper limb functionality in women post-breast cancer surgery? a randomized three-arm clinical trial
Source: Support Care Cancer. 2026 Feb 12;34(3):189. doi: 10.1007/s00520-026-10362-1 (PMC12894169; doi:10.1007/s00520-026-10362-1)

**Material suplementar**

| **Table**  Characteristics of the MoveMama study participants included in the present study in the baseline period, according to the randomized group (n = 69). | | | | | |
| --- | --- | --- | --- | --- | --- |
|  | **Total**  **(n=69)** | **Pilates**  **(n = 25)** | **Dance**  **(n =22)** | **Control**  **(n = 22)** | **p value** |
|  |  | Média (DP) | Média (DP) | Média (DP) |  |
| **Age** ^a^ | 55.4(10.4) | 54.4(10.4) | 55.0(10.0) | 56.8(11.2) | 0.688 |
| **Months after surgery ^d^** | 29.1(18.0) | 29.3(17.3) | 25.4(17.4) | 33.0(19.0) | 0.223 |
|  |  | n (%) | n (%) | n (%) |  |
| **Body Mass Index** ^c^ |  |  |  |  | 0.621 |
| Normal weight (18.5–24.9) | 23(33) | 8(32) | 9(41) | 6(27) |  |
| Overweight (acima 25.0) | 46(67) | 17(68) | 13(59) | 16(73) |  |
| **Surgery** ^c^ |  |  |  |  | 0.073 |
| Mastectomy | 24(35) | 13(52) | 6(27) | 5(23) |  |
| Conservative surgery | 45(65) | 12(48) | 16(73) | 17(77) |  |
| **Surgery side** ^c^ |  |  |  |  | 0.137 |
| Right | 32(46) | 9(36) | 14(64) | 9(41) |  |
| Left | 37(54) | 16(64) | 8(36) | 13(59) |  |
| **Breast reconstruction** ^b*^ |  |  |  |  | 0.303 |
| Yes | 12(52) | 7(58) | 4(67) | 1(20) |  |
| No | 11(48) | 5(42) | 2(33) | 4(80) |  |
| **Axillary approach** ^b^ |  |  |  |  | 0.108 |
| Lymph node dissection | 39(57) | 17(68) | 8(36) | 14(64) |  |
| Sentinel lymph node only | 14(20) | 2(8) | 7(32) | 5(23) |  |
| None | 16(23) | 6(24) | 7(32) | 3(13) |  |
| **Hormone therapy modality** ^c^ |  |  |  |  | 0.259 |
| Inibidores de aromatase | 41(59) | 17(68) | 10(45) | 14(64) |  |
| Tamoxifen | 28(41) | 8(32) | 12(55) | 8(35) |  |
| **Physiotherapy** ^b^ |  |  |  |  | 0.378 |
| Participate | 4(6) | - | 2(9) | 2(9) |  |
| Does not participate | 65(94) | 25(100) | 20(91) | 20(91) |  |
| Note: ^a^ One-Way ANOVA with Bonferroni post hoc. ^b^ Fisher's exact test. ^c^ Chi-square test. ^d^ Kruskal-Wallis test.  * In the breast reconstruction variable, only patients who underwent mastectomy were considered (n = 24).  Missing – Breast reconstruction (n = 1), time after surgery (n = 1). | | | | | |

**Figures**

Sixteen-week belly dance protocol (48 sessions) for women after breast cancer diagnosis (Florianopolis SC, Brazil, 2018).

**
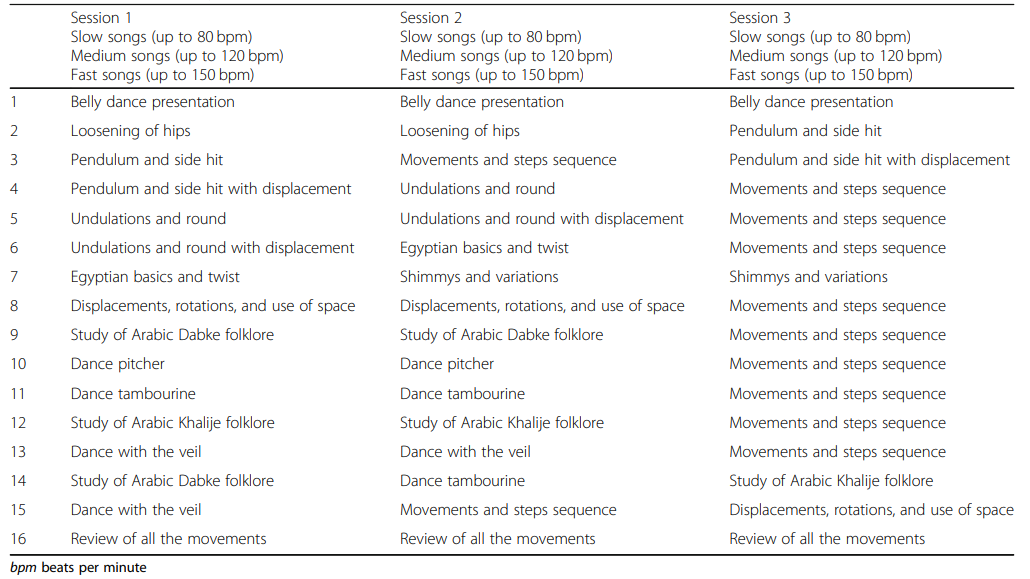
**

Sixteen-week Pilates solo protocol (48 sessions) for women after breast cancer diagnosis (Florianopolis SC, Brazil, 2018).

**
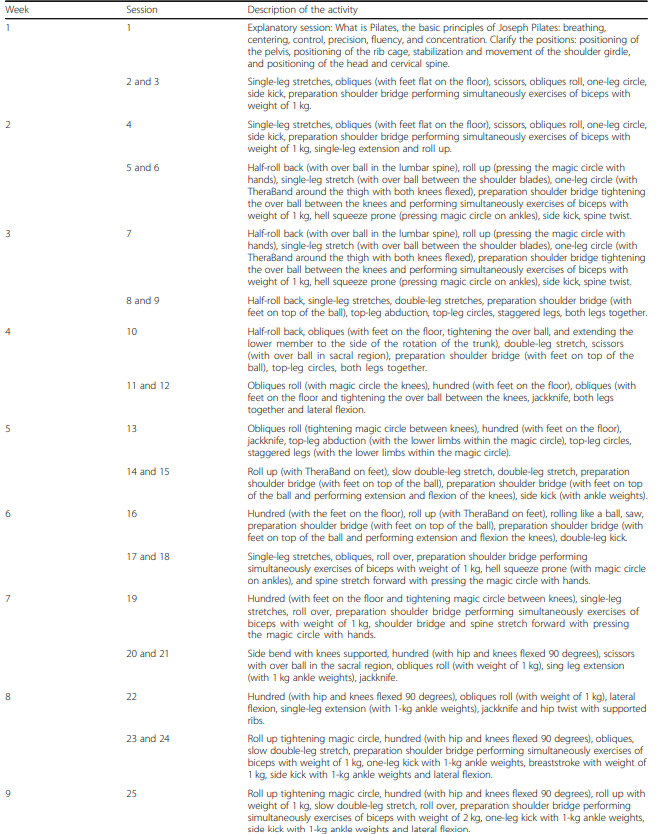
**

Continued - Sixteen-week Pilates solo protocol (48 sessions) for women after breast cancer diagnosis (Florianopolis SC, Brazil, 2018).


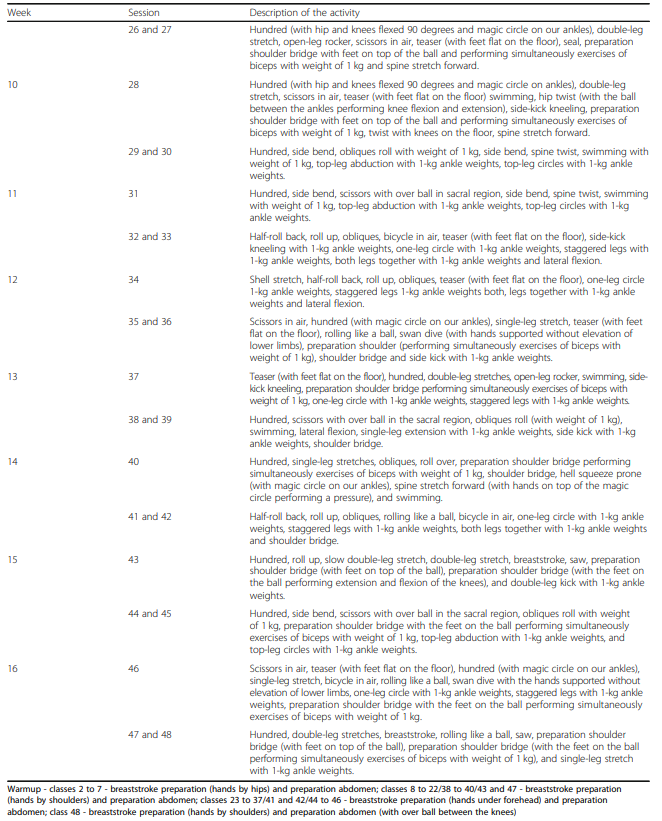

Supplement: Supplementary file 2 — Supplementary file2 (DOCX 571 KB) [file 520_2026_10362_MOESM2_ESM.docx]
